# Correction: Study on ultrasonic leaching and recovery of fluoride from spent cathode carbon of aluminum electrolysis

**DOI:** 10.1039/d3ra90052e

**Published:** 2023-06-15

**Authors:** Chenchen Wang, Song Mao, Longjiang Li

**Affiliations:** a College of Mining, Guizhou University Guiyang 550025 China gzsongm@126.com; b Guizhou Key Laboratory of Comprehensive Utilization of Non-metallic Mineral Resources Guiyang 550025 China; c National & Local Joint Laboratory of Engineering for Effective Utilization of Regional Mineral Resources from Karst Areas Guiyang 550025 China

## Abstract

Correction for ‘Study on ultrasonic leaching and recovery of fluoride from spent cathode carbon of aluminum electrolysis’ by Chenchen Wang *et al.*, *RSC Adv.*, 2023, **13**, 16300–16310, https://doi.org/10.1039/D3RA02088F.

The authors regret that the email address of the corresponding author (gzsongm@126.com) was not shown in the original manuscript. The corrected list of affiliations is as shown here.

The Royal Society of Chemistry apologises for these errors and any consequent inconvenience to authors and readers.

## Supplementary Material

